# Knowledge, attitudes, and practices related to dietary salt among older adults in Abha, Saudi Arabia

**DOI:** 10.1186/s41043-024-00545-z

**Published:** 2024-04-22

**Authors:** Amani Alhazmi, Manal Mohammed Hawash, Haroon Ali, Bayapa Reddy Narapureddy, Farah Aziz

**Affiliations:** 1 Department of Public Health, College of Applied Medical Sciences, King Khalid University, Abha, Saudi Arabia; 2 Department of Gerontological Nursing, Faculty of Nursing, Alexandria University, Alexandria, Egypt; 3 Department of Basic Medical Sciences, College of Applied Medical Sciences, King Khalid University, Abha, Saudi Arabia

**Keywords:** Dietary salt intake, Knowledge, Attitude, Older adults, Practices, Sociodemographic factors

## Abstract

The need to foster successful aging has intensified with the aging of the global population. This study aimed to assess the knowledge, attitudes, and practices (KAP) concerning dietary salt consumption and to investigate the correlations between sociodemographic variables and salt-related KAP. A structured interview was administered to a cohort of 200 older adults in Abha City, Saudi Arabia, recruited through a convenience sampling approach. The evaluation of salt-related KAP revealed widespread low knowledge (91.5%) as participants scored less than 3, negative attitudes (85.5%) scored less than 12, and predominantly unsatisfactory practices (69.5%) with scores less than 26. Noteworthy differences emerged between participants with poor overall KAP (81.5%) and those with good KAP (18.5%). Significantly weak negative correlations were found between age (*r*=-0.212), marital status (-0.236), and body mass index (-0.243) with overall KAP. Further examination revealed a significantly weak positive correlation between attitude and practice (*r* = 0.141). KAP scores show a highly significant positive correlation with overall KAP scores (*r* = 0.169, 0.352, 0.969). The uncovered correlations contribute to a valuable understanding of the complex dynamics surrounding salt-related KAP. This understanding guides the design of targeted interventions, such as health education programs, promoting successful aging and public health outcomes.

## Introduction

Promoting successful aging involves maintaining high physical, psychological, and social functioning in old age without major diseases [1], which has become a growing concern in public health as the population ages. Adapting lifestyle patterns such as dietary modification and physical activity is essential for achieving successful aging later in life [2, 3]. One of the modifiable lifestyle factors that can greatly influence the overall health and successful aging of older adults is dietary salt intake [4]. Dietary salt is an essential micronutrient for supporting human functions, such as blood pressure control, fluid and electrolyte balance, and plasma volume maintenance, facilitating nerve impulse communication, and ensuring optimal cellular and muscle function [5, 6]. The World Health Organization (WHO) suggested that adults cap their daily salt intake at 5 g as a maximum limit [7]. However, a considerable portion of the population surpasses this recommended threshold. The median sodium intake among Saudi adults was reported to be 10.68 g/d, which exceeded the WHO recommendation of 5 g/d [8]. The primary sources of salt in the Saudi diet are often processed and restaurant-prepared foods, which are rich in salt as a flavour enhancer and preservative. Additionally, traditional Saudi dishes contribute to high sodium intake due to the importance of spices and salt [9].

Excessive salt intake is associated with various adverse health outcomes and can particularly affect older adults [10]. High sodium intake can accelerate the aging process through telomere shortening, which enhances cell damage and dysfunction. Age-related renal function also significantly influences the ability of the body to eliminate excess sodium, which results in hypernatremia, decreased medication effectiveness, and central nervous system dysfunction [11, 12]. Salt sensitivity, which refers to the change in blood pressure in response to a decrease or increase in sodium intake, varies from person to person. However, it can have a significant impact on blood pressure, especially in older adults who are already vulnerable to cognitive decline and neurodegenerative diseases that can be linked to excessive salt consumption [13]. An imbalance in sodium levels can influence an individual’s stability and mobility, further exacerbating the existing challenges many older adults face [14]. Excessive salt intake can exacerbate age-related hypertension and cardiovascular diseases (CVD) risk, potentially leading to stroke, heart disease, osteoporosis, and kidney problems [10, 15]. In a study conducted in 2021 by Mumena et al., the sodium intake of 93.5% of the Saudi participants surpassed the daily recommended limit of < 2000 mg set by the World Health Organization [8].

The burden of excess sodium consumption constitutes a significant public health challenge worldwide. Annually, an estimated 1.89 million deaths are attributed to excessive sodium consumption, a well-established factor contributing to hypertension and an elevated risk of CVD [16]. Kalogeropoulos et al. revealed that 35.2% of older adults who consumed more than 2,300 mg of salt died because of heart-related problems [17]. In Saudi Arabia, the economic burden of CVD was estimated to be $3.5 billion, expected to triple to $9.8 billion by 2035 [18]. A recent study in Abha city, Asir region, revealed that ischemic heart disease (IHD) prevalence was 80.7% compared to other CVD. ST-elevation myocardial infarction (STEMI) rate was 37.3%. The unique geographical features of the Asir region, such as high altitude, cool climate, and low oxygen levels, may contribute to the high incidence of CVD [19]. Therefore, lowering sodium consumption is one of the most cost-effective strategies for enhancing health and alleviating the burden of non-communicable diseases (NCDs). A modest 15% reduction in population salt intake might prevent 8.5 million CVD deaths over ten years worldwide, resulting in significant economic savings for individuals, their families, and healthcare systems [17].

It is crucial for older adults to manage their dietary salt intake, as several factors can contribute to increased salt consumption. These factors include lifelong dietary habits, reduced salt taste sensitivity, reliance on processed and convenience foods, lack of awareness of high-sodium foods, cultural dietary practices, social isolation, medication interactions, palatability, family influences, and accessibility of low-sodium options [20]. Personality traits can also influence older adults’ dietary salt intake. One study found that people with personality traits characterized by organization, self-discipline, and goal-directed behavior tended to consume salt in moderation. In contrast, individuals with personality traits characterized by anxiety, mood swings, and emotional instability tended to consume more salt to regulate negative emotions [21]. It is recommended by WHO that individuals’ knowledge and attitudes towards salt intake are evaluated to promote healthy salt-related behaviors [7]. Having knowledge about salt’s potential health risks plays a crucial role in influencing salt consumption practices among individuals. When individuals are informed about the potential health risks associated with excessive salt intake, they are more likely to make conscious choices regarding salt consumption [22]. A study by Hanbazaza, M.A., and Mumena, W.A. (2020) highlighted that Saudi adults have low salt-related knowledge and poor practices towards reducing salt intake [9]. A meta-analysis conducted across twelve high-income countries reported low KAP related to dietary salt intake [20]. According to a study conducted in Malay in 2021, older participants had positive attitudes towards healthy salt intake, but their knowledge and practice related to salt were unsatisfactory [23]. It is crucial to help individuals make informed decisions about their dietary salt intake and reduce excessive consumption for better overall health [6, 8, 9].

Several countries, including Kingdom of Saudi Arabia (KSA), are making efforts to reduce salt consumption. However, it is still important to intensify these efforts to prevent the health consequences of excessive salt intake. The Saudi Ministry of Health has implemented a national strategy to reduce the population’s salt intake by 30% before 2025 [24]. This strategy includes interventions such as menu labeling, awareness campaigns, and product reformulation. The Saudi Food and Drug Authority (SFDA) has also established the Healthy Food Strategy (HFS) as part of KSA Vision 2030, which aims to reduce sodium in 22 processed food items [24, 25]. Despite these initiatives, there is still a lack of attention given to salt-related knowledge, attitudes, or practices (KAP) among older adults in KSA. This is due to the challenges in obtaining reliable self-reporting and reaching the older adult population with mobility or health issues. To address this gap, a study was conducted to assess salt-related KAP among older adults in Abha, KSA. This study aims to develop and implement salt-related awareness programs that promote healthier salt consumption habits and improve health outcomes of older adults in KSA. The research questions of this study were (1) What are the levels of salt-related KAP among older adults in Abha, KSA, and (2) What is the correlation between sociodemographic and clinical factors and salt-related KAP levels?

## Materials and methods

### Sampling and sample size

A convenient, nonprobability sampling technique was used to recruit the study participants. The sample size was determined using Cochran’s formula, given a 95% confidence interval and an estimated proportion of 50% due to the absence of previous studies in similar population [26]. With a margin of error of 7.0%, the calculated total sample size was 200. The study recruited 200 older adults attending primary health care centers and recruited the participants according to the inclusion criteria of being 60 years and above and free from CVD, hypertension, and cognitive impairment. Confirmation of these criteria was obtained from the records of primary healthcare centers, ensuring the selection of eligible participants, among whom 49 were males and 151 were females.

### Data collection

A structured interview was conducted face-to-face with each participant in a comfortable and private setting in primary health centres to establish a rapport and discuss the purpose of the study. The height and weight of the participants were measured three times following established protocols, and the average values were reported. Weight was measured using a portable electronic scale (Omron, BF508, Japan), with measurements recorded to the nearest 0.1 kg. Height was measured with a measuring tape against a straight wall, and measurements were recorded to the closest 0.5 cm. The body mass index (BMI) of each participant was estimated based on their weight and height measurements, and weight status was assessed using WHO criteria. The data were gathered using structured interviews, and each interview lasted an average of 20 min. The survey questionnaire was originally prepared in English and then translated into Arabic by an expert translator to ensure comprehensibility and translation quality. Subsequently, a different multilingual translator translated the questionnaire back into English to confirm that both versions conveyed the same content and meaning. The content and face validity of the questionnaire were rigorously assessed. The Arabic version of the questionnaire was reviewed and subsequently submitted to a panel of five experts from various fields, including nutrition, community medicine, public health, community health nursing, and gerontological nursing. These experts evaluated the face and content validity of the instrument through academic email correspondence, and the panel unanimously agreed that the instrument was explicit, relevant, and legitimate. The questionnaire was pilot-tested with 30 participants from the target population under the same study settings to assess its practical utility and ensure the comprehensibility of the questions. Each interview required approximately ten minutes, and no modifications were required. Responses from the pilot study were excluded from the primary analysis. The Arabic version of the questionnaire was used to assess internal reliability utilizing Cronbach’s alpha correlation coefficient. The findings demonstrated high internal consistency (α = 0.867). Kaiser-Meyer-Olkin Measure of Sampling Adequacy was also done and determined at 0.86. it is above 0.6, which is acceptable. The Bartlett’s Test of Sphericity was done using exploratory factor analysis, and it was significant at 0.0001. hence, the questionnaire construct validity was substantial. Also, checked the reliability and cultural acceptability of the questionnaire.

### Dietary salt KAP questionnaire

A questionnaire containing 34 questions was developed by the researchers based on information obtained from the literature to assess demographic characteristics and KAP related to dietary salt intake. The questionnaire comprises four sections. The first section assessed the sociodemographic and anthropometric profiles of the respondents, such as age, sex, educational attainment, financial and marital status, occupational type, height (centimeters), weight (kilograms), and the presence of chronic illnesses. The second section pertained to the participants’ dietary habits, where they could select from various diet types, including conventional diet**/**ordinary, plant-based diet, and special diets such as diabetic or ketogenic diet. A set of categorical responses was given for each inquiry. The third section consisted of six questions assessing participants’ salt intake knowledge. For the first question, participants were asked, “Do you think high salt intake could affect your health negatively?” They can choose from three responses: yes, no, or I do not know. The second question examines participants’ knowledge about health conditions linked to high salt intake. They are prompted to select from a list of potential conditions, including high blood pressure, kidney disease, heart attack/heart disease, stroke, and stomach cancer. The third question focuses on participants’ understanding of the relationship between salt and sodium. They are presented with options: salt contains sodium, sodium contains salt, or I do not know. For the fourth question, participants are asked about their perception of the primary source of salt in the population’s diet. Options include salt added during cooking or at the table and salt from processed foods such as cheese and breads. The fifth question explores participants’ knowledge of dietary recommendations for salt intake. Salt intake is presented with different amounts: three grams (about 0.5 teaspoons, five grams (about one teaspoon), eight grams (about one and a half teaspoons), and 15 g (about three teaspoons), and asked to choose the quantity they believe healthy professionals recommend. An additional option is provided for those unsure, labelled, as “I do not know.” The final question seek participants’ knowledge on their daily dietary salt consumptions. Response options include excessive, appropriate, insufficient, significantly insufficient, and I do not know. A cut-off points of three indicated low knowledge if less than three points and high knowledge if more than 3 points.

The fourth part of the questionnaire comprises six questions, including attitude statements that measure the participants’ agreement related to salt intake. These statements cover topics such as the need for laws that restrict the quantity of salt added to processed food, the scarcity and limited variety of low dietary salt options when dining out; the difficulty in obtaining salt information on food labels, the potential health benefits of reducing salt intake; the role of added salt in improving food palatability; and the perceived health benefits of Himalayan salt, pink sea salt, and gourmet salts compared to table salt. A five-point Likert scale is used to evaluate various salt-related attitudes, ranging from “strongly disagree” to “strongly agree”. The scores within this section range from six to 18. A cut-off point of 12 was used to indicate a negative attitude if less than 12 points and a positive attitude if more than 12 points.

The last section of the questionnaire comprises twelve items related to salt intake practices. These include salt utilization during the culinary process, the presence of salt on dining tables, the positioning of saltshakers, practices concerned with avoiding fast or packaged food consumption, salt additives during food preparation, eating from Asian-style restaurants, and substituting spices and herbs for salt. Responses were categorized as ‘always,’ ‘often,’ ‘sometimes,’ ‘rarely,’ or ‘never.’ Responses were categorized as ‘always,’ ‘often,’ ‘sometimes,’ ‘rarely,’ or ‘never.’ The scores in this section ranged from a minimum of 12 to a maximum of 42. Participants’ practices were categorized as poor if the scores were less than 26 points, whereas they were categorized as good if the scores were above 26 points. The overall KAP score was 66, categorized as poor if less than 60% and good if 60% or more.

### Statistical analysis

The collected data were entered into Microsoft Excel and analyzed by using Statistical Package for Social Sciences (SPSS) software, version 21.0. Using descriptive analysis, sociodemographic data, and sodium consumption information about participants’ KAP regarding salt intake were examined to determine the frequency, percentage, mean and standard deviation. Comparisons of KAP-related variables were analyzed using an independent t-test, and Spearman’s correlation was used to analyze the relationships between KAP-related variables and sociodemographic variables. However, a relationship was detected via Pearson correlation analysis for the KAP scores. Fisher’s exact and chi-square tests were used to analyze the associations between KAP variables and salt intake and sociodemographic characteristics. *p* < 0.05 indicated statistical significance. Kaiser-Meyer-Olkin Measure of Sampling Adequacy and the Bartlett’s Test of Sphericity were done using exploratory factor analysis to measure construct validity and its level of significance. Also, Cronbach’s alpha was used to check the internal reliability and consistency of the questionnaire.

### Ethical considerations

The study was conducted in accordance with the Declaration of Helsinki and approved by The Research Ethics Committee at King Khalid University (HAPO-06-B-001) with no approval from ECM#2023–2802. To ensure data confidentiality, no personal identifiers were collected during the study. Informed consent was obtained from all participants.

## Results

Table 1 illustrates the sociodemographic and clinical profiles of the 200 participants. Nearly half of the participants were aged 60–69 (47.0%), 34.0% were aged 70–79, and 19.0% were aged 80 years and older. The majority of the participants (75.5%) were female. Regarding educational levels, 74% of the participants were illiterate. Financially, 81.5% reported having enough income. Over half of the participants (52.0%) were single, widowed or divorced. Predominantly, 69.0% were housewives, 22.0% were non-professionals, and 9.0% were professionals before retirement. Regarding BMI, 39.0% were normal, 33.0% were overweight, 23.0% were obese, and 5.0% were underweight. Regarding chronic illnesses, 52.0% of the participants reported no chronic health conditions, while 48.0% reported having at least one chronic illness. The majority of the participants followed an ordinary diet (86.0%), 5.0% were vegetarians, and 9.0% adhered to a special diet.

**Table 1:** Sociodemographic and clinical characteristics of the participants (*n* = 200)

Characteristics	Frequency (No)	Percentage (%)
**Age (Years)**		
60–69	94	47.0%
70–79	68	34.0%
80 and above	38	19.0%
**Gender**		
Female	151	75.5%
Male	49	24.5%
**Educational Levels**		
Illiterate	148	74.0%
Primary education	41	20.5%
Secondary education	11	5.5%
**Financial Status**		
Enough	163	81.5%
Not enough	37	18.5%
**Marital Status**		
Single (Widow, divorced)	104	52.0%
Married	96	48.0%
**Occupation before retirement**		
Housewife	138	69.0%
Nonprofessionals	44	22.0%
Professionals	18	9.0%
**BMI**		
Under weight	10	5.0%
Normal	78	39.0%
Overweight	66	33.0%
Obese	46	23.0%
**Presence of a chronic illness**		
No	104	52.0%
Yes	96	48.0%
**Type of diet**		
Ordinary	172	86.0%
Vegetarian	10	5.0%
Special diet (diabetic diet, Ketogenic diet)	18	9.0%
**Total**	200	100.0%

Body mass index (BMI)

Table 2 shows the mean scores and levels of dietary salt KAP among the participants. Regarding dietary salt knowledge, most participants (91.5%) exhibited low knowledge, which significantly differed from the high-knowledge category (*p* < 0.001). Concerning dietary salt attitudes, the majority of the participants (85.5%) possessed a negative attitude toward salt intake, and this difference was significant (*p* < 0.001). A preponderance of negative practices (69.5%) was observed relative to positive practices (30.5%). Overall, the dietary salt KAP evaluation revealed that 81.5% of the participants were poor, whereas 18.5% demonstrated good overall KAP (*p* value < 0.001).

**Table 2:** Mean scores and levels of dietary salt KAP among the participants (*n* = 200)

Variables	KAP Levels	Average Score (Mean ± S.D)	Frequency (No)	Percentage (%)	*p* value
Knowledge	Low Knowledge	1.73 ± 0.92	183	91.5	0.000*
	High Knowledge	4.24 ± 0.44	17	8.5	
	Overall Knowledge	1.94 ± 1.13	-	-	-
Attitude	Negative Attitude	7.84 ± 1.33	171	85.5	0.000*
	Positive Attitude	12.45 ± 1.38	29	14.5	
	Overall attitude	8.51 ± 2.10	-	-	-
Practice	Poor Practice	15.76 ± 4.41	139	69.5	0.000*
	Good Practice	31.85 ± 4.77	61	30.5	
	Overall Practice	20.67 ± 8.69	-	-	-
Overall KAP	Poor-KAP	27.72 ± 6.09	163	81.5	0.000*
	Good-KAP	46.11 ± 5.72	37	18.5	
	Overall-KAP	31.12 ± 9.35	-	-	--
Total		-	200	100.0	

*Highly significant (*p* < 0.001)

Table 3 displays the correlation between participant characteristics and KAP regarding salt consumption among the participants. The findings reveal several noteworthy associations. Age was weakly negatively correlated with attitude, practice and overall KAP, with *p* values of 0.049, 0.006 and 0.003, respectively. Marital status also exhibited a weak negative significant correlation with attitude, practice and overall KAP (*p* = 0.029, 0.001). In addition, BMI demonstrated a weak negative significant correlation with attitude, practice and overall KAP, with *p* values of 0.005, 0.002, and 0.001, respectively. Notably, educational levels displayed a weak positive correlation with knowledge (*p* = 0.081). Moreover, the presence of a chronic illness demonstrated a weak negative correlation with practice (*p* = 0.010) and overall KAP (*p* = 0.024). The type of diet showed a relatively weak positive correlation with knowledge (*p* = 0.004) and attitude (*p* = 0.010).

**Table 3:** Correlations between sociodemographic variables and knowledge, attitudes, practices, and overall KAP regarding salt consumption (*n* = 200)

Characteristics	Knowledge		Attitude		Practice		Overall (KAP)	
	r	*p*	r	*p*	r	*p*	r	*p*
Age	− 0.061	0.393	− 0.139*	0.049	− 0.192**	0.006	− 0.212**	0.003
Gender	0.016	0.825	0.109	0.124	0.041	0.563	0.082	0.247
Educational levels	0.124	0.081	− 0.018	0.796	− 0.047	0.505	− 0.046	0.513
Financial status	− 0.018	0.803	− 0.118	0.096	− 0.014	0.843	− 0.063	0.374
Marital status	− 0.044	0.536	− 0.154*	0.029	− 0.216**	0.002	− 0.236**	0.001
Occupation before retirement	− 0.011	0.878	− 0.143*	0.043	− 0.006	0.932	− 0.053	0.454
BMI	0.000	0.999	− 0.196**	0.005	− 0.213**	0.002	− 0.243**	0.001
Presence of a chronic illness	0.052	0.467	− 0.008	0.910	− 0.183**	0.010	− 0.160*	0.024
Type of diet	0.202**	0.004	0.181*	0.010	− 0.011	0.879	0.082	0.249

*Correlation is significant at the 0.05 level **Correlation is significant at the 0.01 level, 0 to < 0.25: Weak correlation, 0.25 to < 0.75: Moderate correlation, 0.75 to < 1: Strong correlation, 1: Prefect correlation. Body mass index (BMI)

Table 4 illustrates the correlation between KAP and overall KAP scores regarding salt consumption among the participants. The knowledge scores showed an insignificant correlation with the attitude and practice scores. However, a significantly weak positive correlation was observed with the overall KAP score (*p* < 0.017). The attitude score was significantly correlated with the practice score and overall KAP score (*p* < 0.047, *p* < 0.001). Moreover, the practice score exhibited a highly significant correlation with the overall score (*p* < 0.001).

**Table 4:** Correlations between participants’ knowledge, attitudes, practices, and overall KAP scores regarding salt consumption

KAP Variables	Knowledge Score		Attitude Score		Practice Score		Overall KAP Score	
	r	*p*	r	*p*	r	*p*	r	*p*
Knowledge Score	1	NA	−.034	.638	.059	.409	.168*	.017
Attitude Score			1	NA	.141*	.047	.352**	.000
Practice Score					1	NA	.969**	.000
Overall Score							1	NA

*Correlation is significant at the 0.05 level **Correlation is significant at the 0.01 level

Table 5 shows the associations between KAP and overall KAP scores regarding dietary salt consumption and between the sociodemographic and clinical data of the participants. Significant associations were observed between age groups and KAP and overall KAP score. Patients in the 60–69 age group had significantly greater scores than did the remaining participants (*p* < 0.039, *P* < 0.029; *P* < 0.002; *P* < 0.001). Compared with male participants, female participants had a significantly greater association with negative attitudes (*p* < 0.001). Compared with married participants, single participants were also significantly associated with negative attitudes, poor practice scores and overall KAP scores (*p* < 0.015, *p* < 0.003 and *p* < 0.001, respectively). Similarly, compared with participants in other occupational categories, the housewife participants in these two groups displayed significant associations with negative attitudes and poor practices (*p* < 0.002, *p* < 0.003). BMI categories exhibited significant associations with KAP and overall KAP score (*p* < 0.049, *p* < 0.008, *p* < 0.037, *p* < 0.002, respectively). Additionally, respondents without chronic illnesses were significantly more likely to have poor practices than were those with chronic illnesses (*p* < 0.025). Moreover, type of diet was significantly associated with low knowledge and negative attitude scores compared to other diet categories (*p* < 0.016, *p* < 0.001).

**Table 5:** Association between knowledge, attitude, practice and overall (KAP) related to dietary salt and sociodemographic and clinical data of the participants

Characteristics	Knowledge Scale			Attitude Scale			Practice Scale			Overall Scale		
	Low No. (%)	High No. (%)	*p* value	Negative (%)	Positive (%)	*p* value	Poor (%)	Good (%)	*p* value	Poor (%)	Good (%)	*p* value
Age (Year)												
60–69	82 (41%)	12 (6%)	0.039^*^	75 (37.5%)	19 (9.5%)	0.029^*^	56 (28.0%)	40 (20%)	0.002^*^	64 (32.0%)	31(15.5%)	0.000^*^
70–79	63 (31.5%)	5 (2.5%)		64 (32.0%)	4 (2.0%)		54 (27.0%)	12 (6.0%)		63 (31.5%)	4 (2%)	
80 and above	38 (19%)	0 (0%)		32 (16.0%)	6 (3.0%)		29 (14.5%)	9 (4.5%)		36 (18%)	2 (1%)	
Gender												
Female	136 (68%)	15(7.5%)	0.252	136 (68%)	15 (7.5%)	0.001^*^	105(52.5%)	46(23%)	0.984	120 (60%)	31(15.5%)	0.194
Male	47(23.5%)	2(1%)		35(17.5%)	14 (7%)		34(17%)	15(7.5%)		43 (21.5%)	6 (3%)	
Educational levels												
Read and write	135(67.5%)	13(6.5%)	1.000	125(62.5%)	23(11.5%)	0.535	107(53.5%)	41(20.5%)	0.149	120(60%)	28(14%)	1.000
Primary	38 (19%)	3(1.5%)		35 (17.5%)	6 (3%)		27(13.5%)	14(7%)		34(17%)	7(3.5%)	
Secondary	10 (5.0%)	1 (0.5%)		11 (5.5%)	0 (0.0%)		5(2.5%)	6 (3.0%)		9(4.5%)	2 (1.0%)	
Financial status												
Enough	150 (75%)	13(6.5%)	0.526	143(71.5%)	20 (10%)	0.060	113(56.5%)	50(25%)	0.910	134(67%)	29(14.5%)	0.588
Not enough	33 (16.5%)	4 (2%)		28 (14%)	9 (4.5%)		26 (13%)	11(5.5%)		29 (14.5%)	8 (4%)	
Marital status												
Single	99 (49.5%)	5(2.5%)	0.051	95 (47.5%)	9 (4.5%)	0.015^*^	82 (41%)	22 (11%)	0.003^*^	94 (47%)	10 (5%)	0.001^*^
Married	84 (42%)	12(6.0%)		76 (38%)	20 (10%)		57 (28.5%)	39 (19.5%)		69(34.5%)	27(13.5%)	
Occupation before Retirement												
Housewife	124 (62%)	14 (7%)	0.648	126 (63%)	12(6%)	0.002*	95(47.5%)	43(21.5%)	0.003*	108(54%)	30(15%)	0.147
Nonprofessionals	42 (21.0%)	2 (1.0%)		33 (16.5%)	11(5.5%)		33(16.5%)	11(5.5%)		40(20%)	4(2%)	
Professionals	17 (8.5%)	1(0.5%)		12 (6%)	6 (3%)		11(5.5%)	7(3.5%)		15(7.5%)	3(1.5%)	
BMI												
Under weight	9(4.5%)	1(0.5%)	0.049*	7(3.5%)	3(1.5%)	0.008*	5(2.5%)	5(2.5%)	0.037*	6(3%)	4(2%)	0.002*
Normal	68(34%)	10(5%)		62(31%)	16(8%)		47(23.5%)	31(15.5%)		56(28%)	22(11%)	
Overweight	60(30%)	6(3%)		57(28.5%)	9(4.5%)		51(25.5%)	15(7.5%)		58(29%)	8(4%)	
Obese	46(23%)	0(0%)		45(22.5%)	1(0.5%)		36(18%)	10(5%)		43(21.5%)	3(1.5%)	
Presence of a chronic illness												
No	94(47%)	10(5%)	0.556	87(43.5%)	17(8.5%)	0.440	65 (32.5%)	39 (19.5%)	0.025*	80(40%)	24(12%)	0.083
Yes	89(44.5%)	7(3.5%)		84(42%)	12(6%)		74 (37%)	22 (11%)		83(41.5%)	13(6.5%)	
Type of diet												
Ordinary	161(80.5%)	11(5.5%)	0.016*	153(76.5%)	19(9.5%)	0.001*	120(60%)	52 (26%)	0.750	142 (71%)	30(15%)	0.164
Vegetarian	7(3.5%)	3(1.5%)		5(2.5%)	5(2.5%)		6(3%)	4 (2%)		6 (3%)	4 (2%)	
Special diet.	15(7.5%)	3(1.5%)		13(6.5%)	5(2.5%)		13(6.5%)	5(2.5%)		15(7.5%)	3 (1.5%)	
Total	183(91.5%)	17(8.5%)		171(85.5%)	29(14.5%)		139(69.5%)	61(30.5%)		163(81.5%)	37(18.5%)	

*Significant (*p* < 0.05), **Highly significant (*p* < 0.01); Body mass index (BMI)

## Discussion

This study was conducted to assess KAP related to dietary salt consumption among older adults in Abha, KSA, where hypertension and its multiple complications, including CVD pathology, remain major public health concerns [27]. According to the best of literature review, no published studies have examined salt-related KAP among the older adult population in KSA. The results of the present study indicate that, among the participants, only a mere proportion demonstrated high knowledge (8.5%), positive attitudes (14.5%), good practices (30.5%), and an overall good KAP score of 18.5% concerning dietary salt. Direct comparisons between our study findings and those of prior studies in the region are infeasible due to the scarcity of such studies. However, similar investigations in Malaysia, Australia, and India demonstrated comparable findings. Haron H et al. observed 57.4% mean KAP score with a positive attitude towards lowering their daily salt intake among Malay older adults [23]. Grimes et al. reported that nearly nine in ten Australian adults were aware of high salt consumption risks, and one in three knew the daily recommended salt intake. However, Australians continued to consume high salt (83%) mainly through processed foods (75%). More than half, 58% face challenges in finding the lower-salt menu [28]. Bhattacharya et al. found that most participants exhibiting low salt knowledge (47%) and negative attitudes (95%) [29].

The observed significant differences in knowledge scores align with the disparities noted by previous studies highlighting the impact of educational interventions on salt-related knowledge (from 29 to 42%) and practice (from 26 to 41%) among participants [30]. Consequently, it is imperative for healthcare professionals and policymakers to strategically plan and implement awareness creation programs aimed at reducing salt intake among the older adults. These programs should include personalized counseling sessions conducted at health centers, interactive workshops, and the dissemination of educational materials, including visual aids, infographics, and charts. This multifaceted strategy can contribute significantly to improving the understanding of salt-related issues and fostering healthier practices within the elderly population. The community-based programs must also incorporate family involvement to maximize effectiveness and ensuring a holistic approach that promotes sustained behavior change over time.

The study revealed that individuals who were living alone did not embrace healthier dietary habits and exhibited higher salt intake than did their married counterparts. Evidence from observational studies has indicated that different types of social relationships are important and may influence food consumption. Othman F et al. also made similar observations among Malaysian health staff [31]. A cross-sectional study reported that dietary habits tend to be better among older couples than among single respondents. The results from The Nurses’ Health Study reported that divorced or widowed women have a lower vegetable intake than married women [32]. Matrimonial life might establish consistent meal patterns and reduce the intake of commercially prepared and restaurant foods, which are usually high in sodium [33]. Individuals living alone may encounter challenges related to motivation and culinary skills, impacting their ability to prepare nutritious meals. Additionally, significant life changes such as divorce or widowhood can contribute to emotional distress, influencing dietary choices. Tailored interventions should focus on the provision of straightforward, time-efficient, and health-conscious recipes. Additionally, addressing mental health support in these interventions becomes crucial to comprehensively meet the needs of individuals navigating these life circumstances.

Regarding salt intake practices, knowledge scores showed an insignificant correlation with practice scores, whereas attitude scores demonstrated a significant correlation with practice scores. This finding can be explained by the fact that attitude is a key determinant of behavior. If an individual has a positive attitude towards reducing salt intake, they are more likely to perceive it as a desirable behavior and be motivated to engage in it. On the other hand, if an individual has a negative attitude towards reducing salt intake, they may perceive it as an undesirable behavior and be less motivated to engage in it. Therefore, promoting positive attitudes towards healthy behaviors is an effective way to encourage individuals to adopt and maintain healthy habits. The results of this study contrast with those of an earlier study in which gaps in knowledge and attitudes were documented for only 22.6% of participants, and a significant proportion (67.8%) highlighted negative salt consumption practices [34]. This underscores a pervasive challenge in translating knowledge and attitudes into positive behavioral changes. The outcome of a distinct correlation between negative practices and low overall good KAP is in line with the findings of a study from China in which 70% of participants expressed a desire to lower their sodium intake, yet only 39% confirmed having implemented measures to reduce their sodium intake [35]. Enhancing access to low-sodium foods and promoting behaviors that actively restrict sodium intake are crucial steps in preventing salt-related diseases. A previous study revealed that limited awareness of salt does not directly correlate with the salt intake practices of Saudi adults [9]. Rather, the study emphasizes the intricate interplay of various elements in influencing dietary behaviors and creating an environment that supports access to low-sodium options and encourages behaviors that contribute to reducing sodium intake [9].

The knowledge score demonstrated a negative correlation with the attitude score, suggesting that individuals with higher knowledge did not necessarily exhibit more positive attitudes toward salt consumption. This finding aligns with the nuanced nature of the relationship between factual understanding and personal beliefs. However, the knowledge score revealed a weak positive correlation with the practice score and a significantly weak positive correlation with the overall KAP score. This finding implies that while knowledge may not strongly influence attitudes, it has a slightly more notable association with participants’ actual practices and overall salt-related KAP. The attitude score demonstrated a weak positive correlation with the practice score, suggesting that participants with more positive attitudes tended to engage in better salt consumption practices. This connection underscores the potential impact of attitudes on translating knowledge into tangible behavioral changes. Raising awareness about the benefits of reducing salt intake among older adults can be challenging due to deeply ingrained dietary habits, resistance to change, and low literacy levels. Cultural factors, taste preferences, and the misconception that low-salt diets lack flavor can also make it difficult to encourage healthier eating practices. To overcome these barriers, it’s essential to use personalized communication methods. This can include individual and family counseling, which can help navigate resistance and foster understanding. Healthcare professionals can also play an important role by providing expert guidance and support. By addressing these issues, a successful transition to low-salt diets can be promoted among older adults.

Age group exhibited significant associations with KAP and overall KAP score. Notably, the participants in the 60–69 years age group had higher proportions of better scores than did those aged older than 70 years. This divergence may be attributed to the relatively higher educational level among participants aged 60–69 years than among those aged older than 70 years. This finding aligns with the study by Dickson-Spillmann et al., which emphasizes the role of age in influencing knowledge and practices related to dietary habits [36]. Age and education are significant factors that affect human behavior. To encourage healthy eating habits, children and adolescents should be given priority. Young adults can benefit from information on balanced diets and cooking practices. Middle-aged individuals may need guidance on reading food labels and making informed choices Low literacy levels, multi-morbidity, and poor adjustment can pose challenges for health educators in changing the behavior of the elderly [37]. This highlights the importance of a flexible approach in designing educational programs. Tailoring content, incorporating diverse teaching methods, and promoting a culture of lifelong learning can improve the effectiveness of educational programs for people of all ages within this demographic.

The present study revealed negative correlations between BMI and attitudes and practices, as well as overall KAP, which is in line with findings from Moyinihan PJ et al. featuring the consistent influence of BMI on individuals’ perceptions and behaviors related to salt consumption [38]. Additionally, BMI categories exhibited significant associations with KAP and overall KAP scores. The correlation between BMI and salt-related KAP reinforces the interconnectedness of nutritional awareness and body weight, as highlighted in research by Zhang J et al. in China [35] and Hanbazaza MA et al. in KSA [9]. Other studies conducted by Quaidoo EY et al. and Kanellopoulou A et al. observed that individuals with an inaccurate perception of their body image had a 70% lower likelihood of maintaining a healthy nutritional status compared to those with an accurate body image perception (95% CI: 0.15–0.61) [39, 40].

The significant negative attitude of female participants toward salt consumption depicts gender-based disparity and corresponds with findings from the study by McKenzie B et al., highlighting the need for gender-sensitive approaches in promoting healthier dietary behaviors [41]. Gender-based disparities in attitudes toward salt consumption may arise from a combination of cultural, social, and biological factors. Considering the reasons behind these disparities is essential for effective health promotion efforts. Customized approaches that recognize the unique challenges and influences on each gender can contribute to more impactful health outcomes.

The study outcomes underscore significant associations between participants’ marital status and their attitudes, practices, and overall KAP scores. Individuals who were living alone showed pronounced links with negative attitudes, poor dietary practices, and lower overall KAP scores than did their married counterparts. This emphasizes the impact of marital status in shaping dietary behaviors, as single individuals are more prone to harboring less favorable attitudes and engaging in suboptimal dietary practices. The associations observed imply that marital status plays a pivotal role in shaping attitudes and practices related to salt consumption.

Health status can considerably influence health behaviors, and managing chronic conditions often involves a combination of medical interventions and healthy lifestyle changes, including diet. In the current study respondents without chronic illnesses were significantly more likely to practice poor salt intake than were those with chronic illnesses. Similarly, Choi Ey et al. showed that a more significant majority of major NCD patients had a greater low-salt preference than did the general population; however, the overall trend was not significant [42].

Effective health education is vital for individuals with chronic conditions as it addresses gaps in knowledge, enhances self-efficacy, promotes access to resources, and fosters social support. The present study’s findings could be linked to the educational or counseling interventions that individuals received after being diagnosed with chronic diseases. Similar findings were made by Midhet F et al. study in Quassim region in KSA Chronic illness patients, particularly those receiving health education from primary healthcare centers, show improved dietary practices and increased physical activity across all age groups [43]. In contrast to these results, Oh SW et al. reported significant interactions between urinary sodium excretion and age and between urinary sodium excretion and obesity, abdominal obesity, hypertriglyceridaemia, impaired fasting glucose, and insulin resistance [44]. This unexpected association prompted further investigation into the potential influences of chronic health conditions on dietary practices, as explored by Nasreddine L. et al. [34].

The present findings are relevant to the comprehensive study by Alkhalaf M et al. on salt-related behaviors, reinforcing the intricate interplay between KAP [45]. Additionally, research on health behavior theories by Hanbazaza MA et al. supports the idea that positive attitudes are integral to fostering beneficial practices [9]. The robust correlation between practice and overall KAP is consistent with that of Haron H et al., who emphasized the practical manifestation of knowledge and attitudes related to health-related behaviors [23].

This study has several limitations. The use of convenient sampling methods may introduce selection bias and limit the generalizability of the findings to the broader elderly population. Likert scale responses may lead to varied interpretations, and responses might not perfectly reflect their actual attitudes and practices. A study relying on self-reported data may introduce the possibility of recall bias and social desirability bias, influencing the accuracy of the responses. Future research should consider adopting random sampling methods to minimize selection bias and ensure a more representative elderly population. Using a mix of quantitative and qualitative approaches alongside Likert scales can offer a more comprehensive understanding and reduce interpretational variations. Combining self-reported data with objective measures and longitudinal designs can help minimize recall bias and enhance the validity of responses. Finally, researchers can use diverse data sources and employ advanced statistical techniques to navigate the challenges posed by social desirability bias, leading to more accurate findings.

## Conclusion

The findings of this study provide valuable insights into the complex dynamics that influence participants’ KAP towards salt intake. While the study findings are significant for the population of Abha, KSA, recognizing that these dynamics may vary in other populations. To gain a more nuanced understanding and move beyond mere associations, future studies should focus on establishing causal relationships. Specifically, future studies could address the multifaceted influences identified in the current study, considering the diverse sociodemographic and clinical characteristics of the population. To promote behavior change and align salt intake with dietary guidelines, targeted strategies such as personalized counselling sessions, interactive workshops, and community-based programs should be developed to translate knowledge into practical actions.

## Data Availability

No datasets were generated or analysed during the current study.
